# Saliency-Curated Deep Learning for Predicting Receptor Status in Breast Cancer Brain Metastases

**DOI:** 10.3390/jcm15176501

**Published:** 2026-08-22

**Authors:** Rafail C. Christodoulou, Giorgos Christofi, Constantinos Theofylaktou, Rafael Pitsillos, Iliana Aristokleous, Elena E. Solomou, Evros Vassiliou, Michalis F. Georgiou

**Affiliations:** 1 Department of Radiology, Stanford University School of Medicine, Stanford, CA 94305, USA; rafail99@stanford.edu; 2 Faculty of Electrical Engineering, Mathematics and Computer Science, Delft University of Technology, 2628 CD Delft, The Netherlands; 3 Department of Electrical Engineering, National Technical University of Athens, 15772 Zografou, Greece; theofylaktouc@gmail.com; 4 Bioinformatics Department, Cyprus Institute of Neurology and Genetics, Nicosia 2371, Cyprus; rafaelp@cing.ac.cy; 5 Department of Surgery and Urology, Uppsala University Hospital, 75237 Uppsala, Sweden; iliana.aristokleous@uu.se; 6 Endocrine and Breast Surgery, Department of Surgical Sciences, Uppsala University, 75105 Uppsala, Sweden; 7 Department of Internal Medicine-Hematology, University of Patras Medical School, 26500 Rion, Greece; elenasolomou@hotmail.com; 8 Department of Biological Sciences, Kean University, Union, NJ 07083, USA; evassili@kean.edu; 9 Department of Radiology, Division of Nuclear Medicine, University of Miami, Miami, FL 33136, USA; mgeorgiou@med.miami.edu

**Keywords:** breast cancer brain metastases, convolutional neural networks, deep learning, MRI, estrogen receptor, progesterone receptor, HER2, explainable AI

## Abstract

**Background**: Breast cancer brain metastases (BCBMs) exhibit notable receptor discordance between primary tumors and metastases, yet obtaining intracranial biopsies is rarely practical. We created an interpretable deep learning radiogenomic model to predict estrogen receptor (ER), progesterone receptor (PR), and human epidermal growth factor receptor 2 (HER2) status from MRI scans in BCBM cases. **Methods**: A total of 241 post-contrast T1-weighted MRIs from 142 patients were analyzed. We developed a mask-free 3D Residual Neural Network (ResNet) ensemble trained directly on cropped, bias-corrected brain images, with comprehensive 3D geometric and intensity augmentations. The model was optimized in a multi-label setting using Asymmetric Focal Loss. Hyperparameters were fine-tuned via Bayesian optimization, and class probabilities were calibrated. Additionally, Integrated Gradients (IG) offered voxel-level saliency maps. **Results**: Our ResNet ensemble model achieved a micro-averaged AUROC of 0.74 and a macro-averaged AUROC of 0.67. Receptor-specific AUROCs were 0.57 for ER, 0.79 for PR, and 0.64 for HER2. The F1-scores were 0.56, 0.62, and 0.88 for ER, PR, and HER2, respectively. The held-out cohort contained only four HER2-negative patients, so threshold-dependent HER2 metrics are strongly prevalence-driven and AUROC is the more appropriate summary. Qualitatively inspected saliency maps were concentrated on enhancing metastases with limited background attribution. **Conclusions**: This proof-of-concept study shows that a segmentation-free, interpretable 3D Convolutional Neural Network (CNN) can be trained to capture receptor-associated patterns in post-contrast T1-weighted MRI of BCBMs. Accuracy was modest relative to published radiomics models, and the mask-free, single-sequence design is offered as a methodological contribution rather than a performance gain. These findings are preliminary, do not establish clinical utility, and require validation in larger, multi-center cohorts.

## 1. Introduction

Breast cancer is the second most frequent cause of metastasis development in the brain. Brain metastases occur in up to 32% of women with metastatic breast cancer [1,2]. The risk of developing brain metastases and the subsequent patient prognosis vary widely according to breast cancer molecular subtype. The ER, PR, and HER2 act as surrogate markers to estimate these subtypes and to guide treatment decisions and overall management [3]. The incidence of brain metastases is highest in HER2-positive (31%) and triple-negative (32%) metastatic breast cancers, compared with 15% in hormone receptor-positive/HER2-negative disease [4]. PR- or ER-positive breast cancers may respond to endocrine therapy, while HER2-positive breast cancers may benefit from targeted antibody therapies with central nervous system (CNS) activity. Triple-negative tumors, due to their aggressive nature, often require complex chemotherapy regimens [5,6]. Hence, accurate identification of receptor status in the BCBM is essential for personalized treatment and outcome prediction.

However, in clinical practice, the receptor status of BCBMs is rarely confirmed histologically. Intracranial biopsy or surgical resection is performed only in selected cases due to procedural risks and limited feasibility [7]. Even when tissue is available, spatial heterogeneity and sampling bias may lead to incorrect results [8,9]. Clinicians thus rely on the receptor profile of the primary breast tumor because it is more readily accessible. This approach seems inferior, as mounting evidence of receptor discordance has been reported between the primary and metastatic sites. A recent meta-analysis found receptor discordance rates of approximately 17% for ER, 23% for PR, and 12% for HER2, leading to subtype changes in nearly 40% of patients [10]. The most common shifts involved loss of ER or PR and gain of HER2 in metastatic lesions [10]. However, the implications of overall tumor subtype switching and its impact on clinical management are often overlooked in clinical practice [10]. These limitations express the need for non-invasive, reproducible tools to predict receptor status.

Radiogenomics, which integrates imaging and molecular data, is a promising approach. Quantitative MRI features can reflect tumor heterogeneity and microenvironmental differences linked to receptor expression. Previous studies have explored this possibility, with radiomics models predicting receptor status in BCBM with encouraging accuracy, reinforcing the notion that imaging characteristics capture clinically meaningful molecular information [11,12]. Nevertheless, most radiomics pipelines require extensive preprocessing and handcrafted feature extraction, thereby restricting generalizability and scalability [13].

DL advances have reshaped medical imaging analysis by deploying convolutional neural networks (CNNs) to learn complex image representations automatically. CNN-based radiogenomics has been applied to the prediction of genomic and molecular markers across multiple cancers, including gliomas and breast cancer [14,15]. Integrating these models with explainability tools can enhance transparency and build clinician trust, both of which are essential for clinical implementation.

In this study, we developed an explainable DL radiogenomic model for non-invasive prediction of ER, PR, and HER2 receptor status in BCBM. Using the Breast Cancer Brain Metastases dataset, we trained a CNN to learn receptor-associated imaging patterns from post-contrast T1-weighted MRI [16]. The model incorporates explainability via Integrated Gradients (IG) to provide voxel-level region attribution for each prediction, enabling visual assessment of biological plausibility. Instead of relying on manual lesion masks, the model was trained end-to-end to learn spatially relevant patterns only on preprocessed whole-brain MRI volumes, achieving a mask-free workflow that operates on a single modality. The intended contribution is methodological rather than a claim of superior predictive accuracy relative to multisequence radiomics pipelines: the framework removes the manual lesion-delineation step and the multimodal acquisition requirement, thereby reducing expert annotation burden. This approach provides a preliminary framework for investigating receptor-associated patterns using mask-free DL and saliency-based post hoc attribution. If validated in substantially larger and independent cohorts, such an approach could eventually support non-invasive receptor assessment when tissue sampling is unavailable or impractical, and allow timely therapeutic adjustments, in patients with BCBM. To our knowledge, no previous research has presented an MRI-based DL model for predicting ER, PR, and HER2 status in BCBM. Existing studies have primarily used radiomics combined with traditional machine learning (ML) classifiers [11,17].

## 2. Materials and Methods

### 2.1. Data Acquisition

#### 2.1.1. Dataset

For this project, we use the Cancer Imaging Archive (TCIA) Breast Cancer Brain Metastasis (BCBM) dataset [16], which comprises 268 post-contrast T1-weighted brain MRIs from 165 patients with histologically confirmed metastatic breast cancer. The scans were acquired on 1.5 T and 3 T scanners for stereotactic radiosurgery (SRS) planning, with isotropic voxel spacings ranging from 0.5 mm to 1.0 mm. Each image is provided skull-stripped, atlas-aligned, and intensity-inhomogeneity-corrected using N4 bias correction. The dataset also includes clinical metadata (age, sex, and receptor status: ER, PR, HER2) and expert-reviewed segmentation masks of tumors and postoperative cavities. ER, PR, and HER2 status are provided in the dataset metadata as a single patient-level record. As described in the dataset documentation, histologic receptor status was obtained in one of several ways, namely lymph node biopsy, sampling of the primary breast tumor, or biopsy of a metastatic site that could be intracranial or extracranial. The source tissue is therefore heterogeneous across patients and is not necessarily the brain metastasis itself. Lesion-specific receptor annotations are not provided, so every scan belonging to a given patient inherited the same ER, PR, and HER2 label. The prediction task addressed here is consequently the estimation of patient-level receptor status from an individual MRI volume, rather than the estimation of receptor expression within a specific intracranial lesion. However, these annotations were created for broader lesion and treatment-related characterization and were not consistently aligned with the lesion-focused supervision required for our receptor prediction task. Preliminary attempts to identify and retain only task-relevant contours remained sensitive to annotation ambiguity and filtering criteria. We therefore adopted a mask-free design that relies on a single preprocessed MRI modality and avoids dependence on manual lesion delineation. Our final analysis cohort consisted in total of 142 females between the ages of 27 and 88 (57.2 mean age) who were diagnosed with BCBM.

The original TCIA cohort contained 268 scans from 165 patients. After exclusion of 27 scans from 23 patients without available ER, PR, or HER2 labels, 241 scans from 142 patients remained for analysis (Table 1). Among the remaining 241 usable scans, some are missing one or two receptor labels, but at least one label is present for each.

#### 2.1.2. Train–Test Split

**Figure 1:**
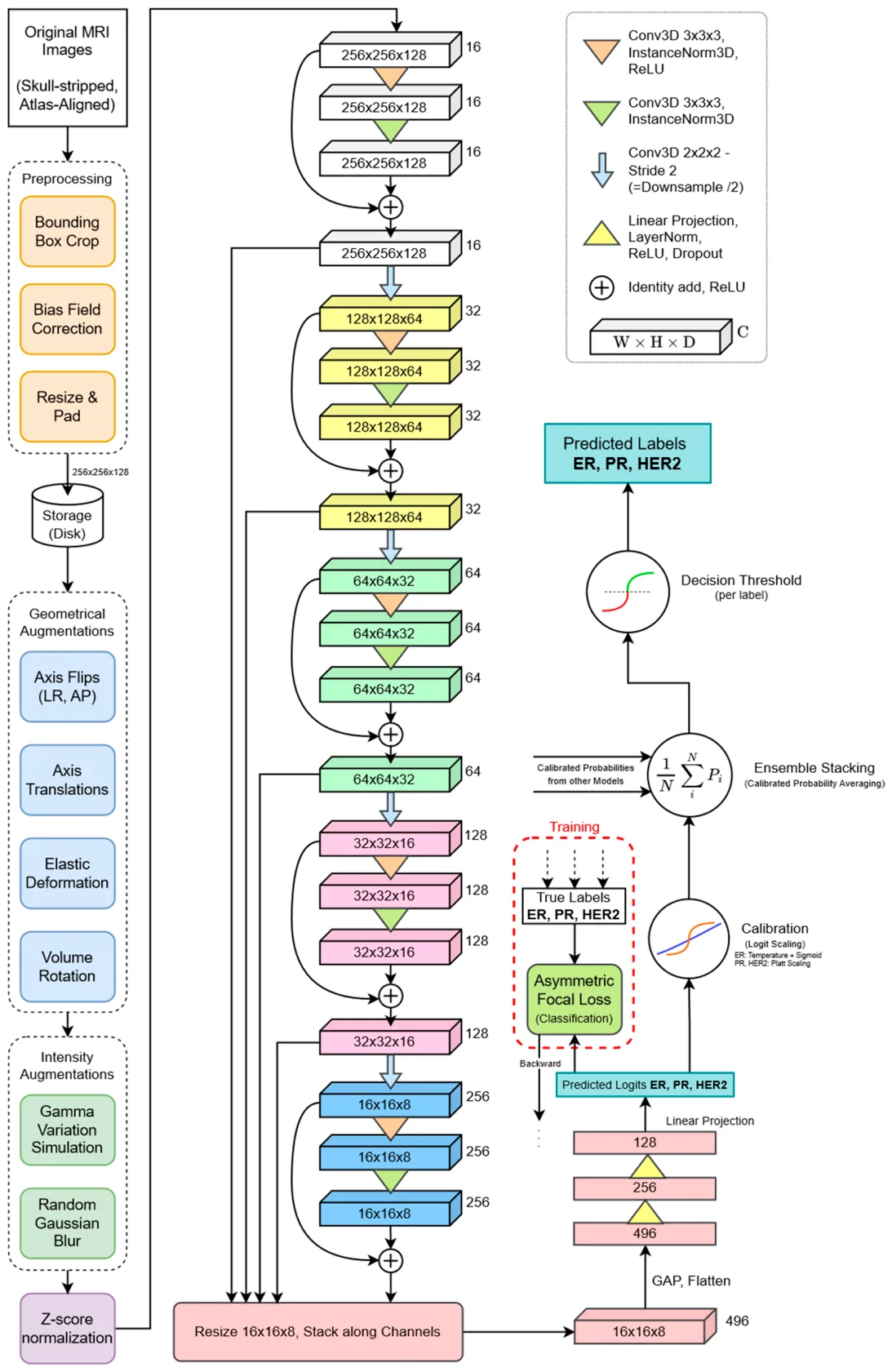
**Complete training and inference pipeline for 3D CNN ensemble in breast cancer receptor prediction.** Skull-stripped MRI volumes undergo preprocessing followed by online geometric and intensity augmentations, and finally intensity normalization (z-score). A multi-scale 3D CNN encoder extracts hierarchical features through progressive downsampling; bottleneck features are spatially resized, concatenated across channels, globally pooled, and linearly projected to produce logits. These logits are then calibrated, sigmoid-activated, and thresholded to yield class predictions. Models are trained using the Asymmetric Focal Loss (ASL), and the final ER, PR, and HER2 labels are obtained via probability averaging and per-class thresholding across the ensemble.

To ensure a valid evaluation and prevent patient-level data leakage, only samples with complete receptor labels (ER, PR, and HER2 jointly available) were eligible for testing. Approximately 10% of the dataset’s usable samples (24/241 scans) were reserved for the held-out test set. Test-set construction was performed at the patient level: 24 unique patients with single scans were randomly selected, and their corresponding MRI scans were assigned exclusively to the test split. Consequently, no patient in the test set appeared in the training or validation data. Of the remaining 217 samples, 17 contained at least one missing label and were therefore assigned directly to the training split. The remaining 200 fully labeled samples were used to perform an internal 5-fold stratified split at the scan level, yielding five validation folds (40 samples each, 20%) with no missing data for cross-validation, hyperparameter tuning, and model selection. These validation folds were also used for model calibration but did not directly influence the neural network parameters; they were used solely for meta-training based on out-of-fold (OOF) predictions. The held-out test set was not accessed during model weight training, calibration, hyperparameter optimization, threshold estimation, or ensemble candidate selection. The proposed framework (Figure 1) consists of three main stages: (i) MRI preprocessing and augmentation, (ii) 3D ResNet-based feature encoding, and (iii) multi-label classification. For our implementation, we used Python 3.10 (Python Software Foundation, Beaverton, OR, USA; https://www.python.org/) and the PyTorch library (version 2.7.1 + CUDA 12.8 build, PyTorch Foundation; https://pytorch.org/) [18].

### 2.2. Model Architecture and Processing Pipeline

#### 2.2.1. Volume Preprocessing

TCIA supplied skull-stripped, atlas-registered, and N4 bias-field-corrected images. All analyzed scans were acquired on Siemens systems; among the 241 included scans, 220 were acquired at 1.5 T, 19 at 3 T, and field-strength information was unavailable for 2 scans. In-plane pixel spacing was 0.5 × 0.5 mm for 240 scans, while one scan had approximately 1 × 1 mm spacing.

Our pipeline subsequently applied additional N4 bias-field correction, cropped each volume around a tight bounding box enclosing the brain, and resized all volumes to the target shape of 256 × 256 × 128 (W × H × D) to preserve model capacity. No dedicated scanner-domain harmonization method was applied.

#### 2.2.2. Data Augmentation and Normalization

Data augmentation was performed during training to increase dataset diversity, improve generalization, and reduce overfitting while maintaining anatomical plausibility. Augmentations were applied probabilistically (with probability *p*) to allow diverse combinations. Geometric transformations included axis flips along the Left–Right (LR) and Anterior–Posterior (AP) axes (RandomFlip3D, *p* = 0.5), axis translations with maximum shifts of 5 (IA), 10 (AP), and 10 (LR) voxels along each axis (RandomTranslation3D, *p* = 0.8), elastic deformations with α = 2.0 and σ = 8.0 (ElasticDeformation3D, *p* = 0.2) [19] and volume rotations with a maximum angle of 8° (RandomRotation3D, *p* = 0.3). Intensity adjustments included gamma variations in the range 0.8–1.2 (RandomGamma3D, *p* = 0.7) and Gaussian blurring with µ = 0 and σ = 0.5 (RandomGaussianBlur3D, *p* = 0.3). The augmentation ranges were intentionally kept conservative to preserve anatomical plausibility while introducing moderate spatial and intensity variability.

Following augmentation, voxel intensities were normalized per volume using z-score standardization.

#### 2.2.3. Encoder Architecture

Feature extraction was performed using a five-layer 3D ResNet encoder [20]. Each block consisted of two consecutive 3 × 3 × 3 convolutional layers, each followed by Instance Normalization and ReLU activation, with an additive identity shortcut connection before the final block activation. Spatial downsampling was performed between successive blocks using a 2 × 2 × 2 convolution with a stride of 2, halving the spatial resolution and doubling the number of feature channels.

Unlike the canonical ResNet configuration, all intermediate block outputs were also directly propagated to the bottleneck, spatially resized to 16 × 16 × 8, and concatenated along the channel dimension. This operation yielded a composite multi-scale representation of size 16 × 16 × 8 × 496, enabling joint modeling of local and global contextual features while facilitating more direct gradient flow during optimization.

#### 2.2.4. Classification Head

The aggregated feature tensor was subsequently subjected to global average pooling (GAP) [21] and passed to a three-layer multilayer perceptron (MLP) classifier. Each layer consisted of a linear projection, Layer Normalization, ReLU activation, and Dropout regularization to mitigate overfitting, given the limited training data. The final layer produced logits corresponding to the three molecular markers of interest: estrogen receptor (ER), progesterone receptor (PR), and HER2 status.

### 2.3. Model Development

Training was conducted under a multi-label setting using the Asymmetric Focal Loss (ASL) [22], which modulates the contribution of positive and negative samples to mitigate class imbalance and improve sensitivity to underrepresented labels. Model parameters were optimized using the AdamW optimizer [23] with a mini-batch size of 4.

Hyperparameter optimization (Table 2) was performed using Optuna’s Bayesian optimization framework [24]. The tuned hyperparameters included the ASL focusing and shifting factors, the optimizer’s learning rate and weight decay, and the classifier-MLP’s dropout probability. We report the selected hyperparameters of the trial, which resulted in the best-performing models.

The model selection objective was defined as a weighted composite score jointly evaluating model discrimination, separability, and stability. Specifically, the objective incorporated the following AUC and Youden’s J index [25] components:

- **Mean validation AUC**: Overall discriminative ability (w = 0.35);
- **Minimum validation AUC**: Prevention of class underperformance (w = 0.35);
- **Mean Youden’s J index (TPR–FPR)**: Positive–negative separability (w = 0.20);
- **Minimum Youden’s J index**: Prevention of class under-separability (w = 0.10);
- **AUC standard deviation penalty**: Consistent performance across classes (w = −0.05);
- **Youden’s J standard deviation penalty**: Consistent separability across classes (w = −0.05).

Each component contributed to a weighted composite score balancing discriminative power, robustness, and inter-class stability.

**Figure 2:**
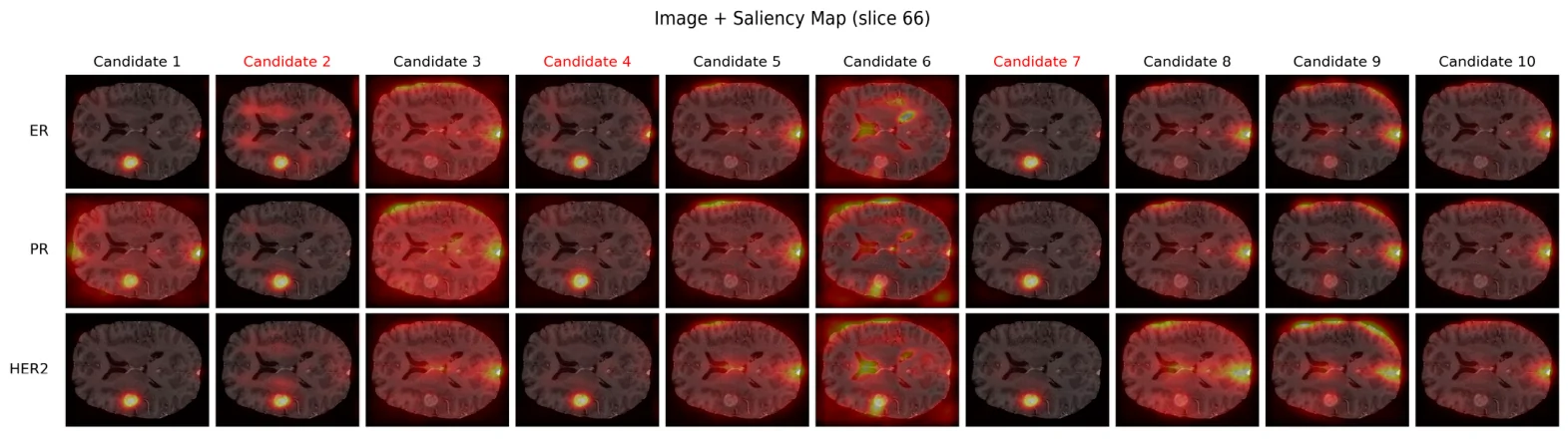
**Slice 66 of case BCBM-RadioGenomics-104-0 shows overlayed, smoothed saliency gradient maps for each class’s output with respect to input voxels across ten candidate models.** The selected models, marked in red, are displayed. From the top-performing validation models, three were selected for the final ensemble based on a combination of validation performance and qualitative assessments of saliency-map plausibility. The selected models focus on brain metastasis with minimal attention to the surrounding parenchyma. The excluded models attributed signals to the superior sagittal sinus, which appears as BM on post-contrast T1, causing them to focus on anatomically implausible regions.

The top 10 performing models across all epochs, folds, and trials were retained as candidate models based on validation performance. Candidate selection for the final ensemble incorporated both quantitative and qualitative criteria. Quantitatively, the selected models demonstrated strong validation performance, with composite validation scores of 0.6431, 0.6688, and 0.6384, respectively. Qualitatively, we additionally inspected Integrated Gradients saliency maps [26] on representative validation samples to exclude models exhibiting anatomically implausible attribution patterns or excessive attention to non-relevant background structures (Figure 2).

We did not employ lesion-overlap metrics between saliency maps and segmentation masks as a formal ensemble selection criterion because the objective of this study was receptor-status prediction rather than lesion localization. The available segmentation masks primarily delineate enhancing metastases on post-contrast T1-weighted imaging; however, receptor-associated imaging signatures may not be confined to the enhancing tumor core. Relevant information may be present in the peritumoral interface or adjacent brain parenchyma, including edema-related, microstructural, or textural patterns that are better characterized by FLAIR, T2-weighted imaging, or DWI/ADC. Given the limitation of using a single T1CE sequence, restricting the CNN to the enhancing lesion mask could have further reduced the available biological signal and limited feature discovery. Therefore, we allowed the model to learn from the cropped whole-brain T1CE volume rather than forcing feature extraction within neuroradiologist-defined enhancing-lesion boundaries. Saliency maps on validation samples were instead used qualitatively to exclude models with clearly implausible attention to irrelevant structures, while preserving the possibility that the CNN could identify subtle spatial or intensity patterns not readily visible to human observers.

Each model was calibrated on its validation fold to align the predicted probabilities with observed outcomes. Two calibration strategies were applied: (i) for ER, logits were scaled with a learnable temperature parameter and converted to probabilities via a sigmoid activation; (ii) for PR and HER2, Platt scaling [27] was used. The scaling parameters were optimized per class by iteratively minimizing the Binary Cross-Entropy (BCE) loss using PyTorch’s implementation of the Limited-Memory Broyden–Fletcher–Goldfarb–Shanno (L-BFGS) optimizer [18], which is well-suited for the small-dimensional convex problems involved in temperature and Platt scaling. Per-class decision thresholds were then determined by linearly scanning the range [0.1, 0.9], and selecting the value that maximized the F1-score.

During inference, each component model produced calibrated probabilities independently, which were subsequently aggregated to create the ensemble’s final prediction.

## 3. Evaluation Results

The ensemble model’s performance was evaluated on a held-out test set of 24 samples, each from a unique patient with a single available scan, with complete receptor labels, ensuring no data leakage from the training and calibration processes.

### 3.1. Classification Performance Metrics

We computed per-class diagnostic performance metrics, including accuracy, precision/positive predictive value (PPV), recall/sensitivity, specificity, negative predictive value (NPV), F1-score, and AUROC, to evaluate the ensemble model on the held-out test set. Results are summarized in Table 3.

In addition to discrimination-based metrics such as AUROC, we report clinically interpretable diagnostic measures, including Positive and Negative Predictive Value (PPV, NPV) at the selected operating thresholds. For HER2 prediction, the ensemble achieved a PPV of 85.7%, indicating that among cases classified as HER2-positive at the selected threshold, 85.7% were HER2-positive in the held-out test cohort. Conversely, the HER2 NPV was lower (33.3%), reflecting the limited number of HER2-negative samples in the test set.

**Figure 3:**
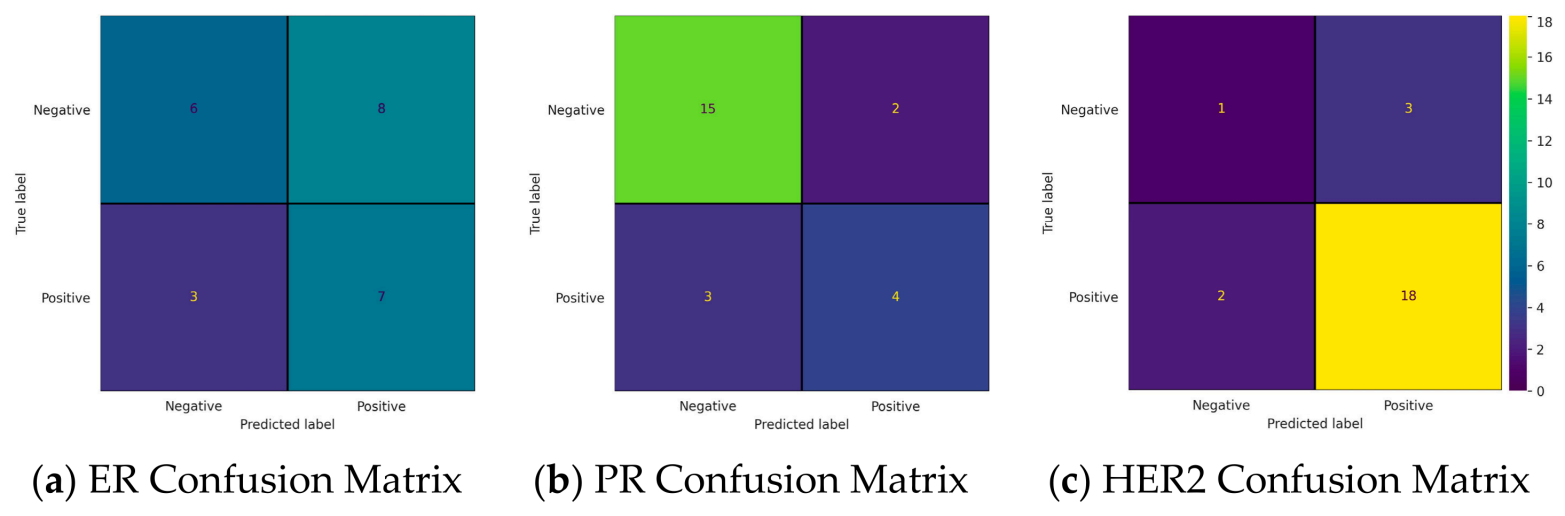
Confusion matrices for ER, PR, and HER2 receptors predicted by the ResNet3D ensemble.

**Figure 4:**
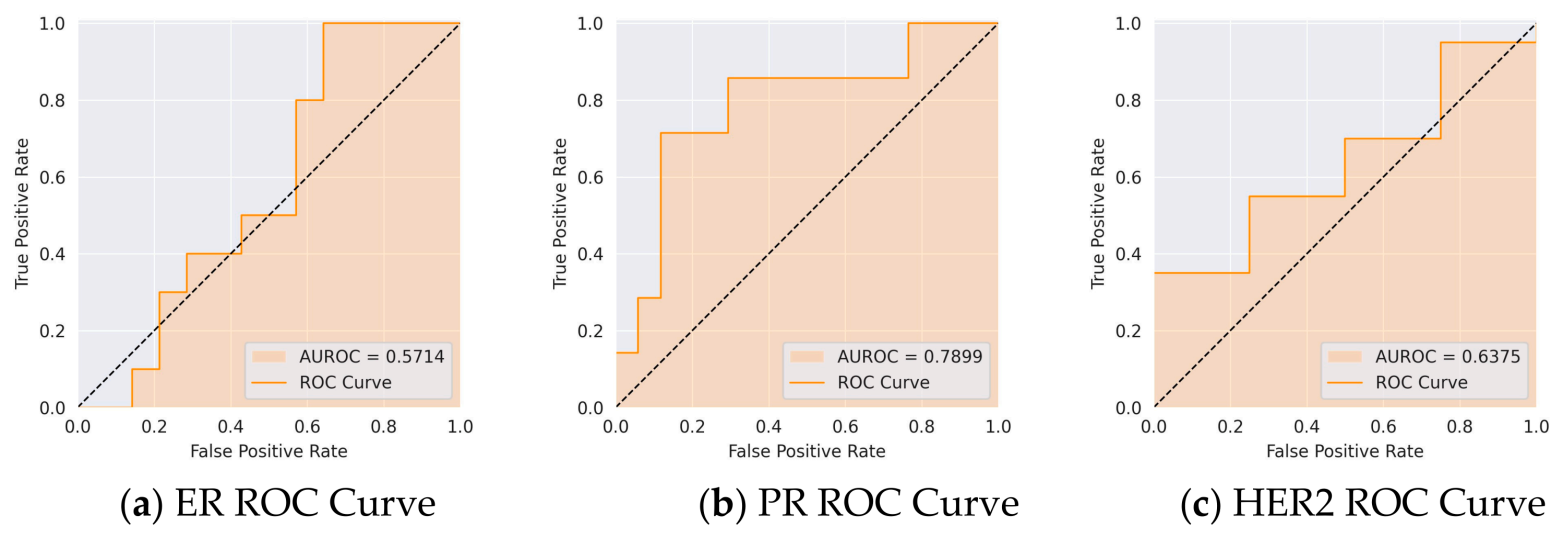
Receiver Operating Characteristic (ROC) Curves and corresponding AUROC values for ER, PR, and HER2 receptors predicted by the ResNet3D ensemble.

To further evaluate the ensemble model’s performance on the test set, we display the per-class confusion matrices (Figure 3) and Receiver Operating Characteristic (ROC) curves (Figure 4).

### 3.2. Analysis

#### 3.2.1. ER

ER prediction showed the weakest overall diagnostic performance among the three receptor targets. The AUROC of 0.5714 indicates poor discrimination between ER-positive and ER-negative cases. Although sensitivity was relatively high (0.7000), the PPV remained low (0.4667), indicating that a substantial proportion of cases predicted as ER-positive were false positives. Specificity was also limited (0.4286), suggesting limited ability to correctly identify ER-negative cases. The associated confidence intervals were relatively wide across several metrics, reflecting substantial uncertainty and limited statistical stability in the estimated ER performance. Taken together, these findings indicate that the current T1CE-only model does not discriminate ER status to a useful degree in its present form.

#### 3.2.2. PR

PR prediction demonstrated the strongest overall diagnostic discrimination among the three receptor targets. The model achieved an AUROC of 0.7899 and an accuracy of 0.7917, indicating the highest observed separability among the three targets. PPV (0.6667) and sensitivity (0.5714) were moderately balanced, while specificity remained high (0.8824), indicating that relatively few false-positive predictions were observed among PR-negative cases. Despite the class imbalance in the test cohort (PR-positive: 29.2%), the AUROC suggests that the model showed stronger discrimination for PR than for ER or HER2 in this cohort. Compared with the other receptor targets, PR-related confidence intervals were also relatively more stable, supporting comparatively greater consistency of the observed diagnostic behavior, although uncertainty remained substantial due to the limitations of the cohort size.

#### 3.2.3. HER2 Receptor

HER2 performance was modest, and interpretation of threshold-based metrics is limited by the very small HER2-negative subgroup in the held-out cohort. At the selected operating threshold, the model achieved a PPV of 0.8571 and sensitivity of 0.9000. However, the AUROC of 0.6375 indicates only modest overall discrimination between HER2-positive and HER2-negative cases across thresholds. This discrepancy should be interpreted in the context of strong class imbalance in the test cohort (HER2-positive: 83.3%), in which threshold-dependent diagnostic metrics may remain favorable despite limited ranking performance. In addition, confidence intervals for specificity and NPV were particularly wide, reflecting the very limited number of HER2-negative cases (n = 4) and reducing the statistical stability of negative-class performance estimates. Consequently, although the model demonstrated high sensitivity at the selected threshold for detecting HER2-positive disease, its ability to reliably exclude HER2-negative cases remains uncertain.

#### 3.2.4. Performance Across All Targets

Across the three receptor prediction tasks, the ensemble demonstrated heterogeneous diagnostic performance profiles. PR prediction achieved the strongest overall discrimination, with an AUROC of 0.7899 and specificity of 0.8824, representing the strongest observed discrimination among the three targets. HER2 prediction achieved a PPV of 0.8571 and sensitivity of 0.9000, although its moderate AUROC (0.6375) suggests modest threshold-independent discrimination. HER2 metrics should be interpreted cautiously given the very small HER2-negative subgroup. ER prediction showed the weakest performance, with an AUROC of 0.5714, PPV of 0.4667, and specificity of 0.4286. Confidence intervals were generally wider for metrics associated with minority classes, particularly HER2-negative cases, reflecting the limited size and class imbalance of the held-out cohort. The HER2-positive fraction of the held-out cohort (83.3%) also exceeded that among scans with known HER2 status in the full analysis cohort (61.6%), which further limits the interpretability of threshold-dependent HER2 metrics. Consequently, the reported results should be interpreted as preliminary and hypothesis-generating rather than definitive estimates of clinical diagnostic performance.

#### 3.2.5. Code Availability

All code supporting the findings of this study is openly accessible at the following GitHub repository: https://github.com/Const1357/BCBM-RadioGenomics-Classifier.git (accessed on 25 June 2026).

## 4. Discussion

Our interpretable 3D CNN ensemble showed variable discrimination across the three receptor targets in a small, patient-independent held-out cohort. Discrimination was highest for PR (AUROC 0.79), modest for HER2 (AUROC 0.64), and close to chance for ER (AUROC 0.57). All three estimates carry wide confidence intervals, and none should be read as an established level of diagnostic performance. For HER2 in particular, the threshold-dependent metrics are an unreliable summary in this cohort. The held-out set contained 20 HER2-positive and only 4 HER2-negative patients, so a trivial classifier labeling every case HER2-positive would already reach 83.3% accuracy. The observed accuracy (0.79) and PPV (0.86) are therefore strongly influenced by prevalence and should not be interpreted as evidence of strong discriminative ability. AUROC is the appropriate summary in this setting, and at 0.64 it indicates only modest ranking performance. We accordingly interpret HER2 discrimination as modest and statistically uncertain. The same caution applies to the ER and PR estimates, which rest on 24 patients in total.

The reason our discrimination differed across the three receptors cannot be determined from these data. The study was not designed to test biological mechanisms, and a 24-patient cohort cannot support attribution of performance differences to tumor biology. The explanations that follow are drawn from previously published work and are offered as hypotheses for future testing, not as findings of the present study. Prior imaging series have described HER2-positive metastases as showing more aggressive growth and more prominent vasogenic edema [17]. Others have reported greater intratumoural heterogeneity in HER2-positive breast cancer than in hormone receptor-positive disease [28]. Whether either property influenced our model’s behavior was not evaluated here and remains unknown.

The comparatively higher PR AUROC observed in this cohort likewise has no mechanistic explanation that our data can support. Published work describes PR-positive metastatic breast cancer as biologically less aggressive, with lower proliferation rates and more favorable survival [29]. Whether a more homogeneous imaging phenotype follows from this was not tested here. ER discrimination was the weakest observed (AUROC 0.57), and several non-exclusive explanations are plausible. First, the ER-associated phenotype may be incompletely represented on post-contrast T1-weighted imaging alone, since ER-positive and ER-negative metastases can show similar enhancement patterns and lesion morphology on this sequence [11]. Second, the sequences reported to be most informative for hormone-receptor status were unavailable to our model. Heitkamp et al. found that their classifier attributed 45% of its reliance to FLAIR-derived features, against 29% for T1CE [17]. Multiparametric radiomics studies have similarly identified FLAIR- and T2-derived texture features within receptor-predictive signatures [11]. Diffusion metrics may carry further complementary information, as lower ADC histogram values have been reported in ER/PR-positive than in ER/PR-negative metastases [30]. Third, the number of ER-labeled patients available for evaluation was small, so the estimate itself is imprecise. The low ER AUROC should therefore not be read as evidence that ER status lacks an imaging correlate. It indicates only that such a correlate was not recoverable from a single sequence in a cohort of this size, and that larger cohorts with multiparametric input are required to test the question properly.

The contribution of this work is methodological rather than a claim of improved predictive performance. Previous radiomics models for this task report higher figures than ours: Luo et al. reported test AUCs of approximately 0.87–0.89, and Heitkamp et al. reported AUROCs of approximately 0.73–0.82 [11,17]. Our ensemble achieved a micro-averaged AUROC of 0.74, with receptor-specific values ranging from 0.57 to 0.79. We do not present our framework as superior to these approaches. What differs is the input requirement. Those pipelines depend on manual lesion segmentation, multisequence acquisition, handcrafted feature extraction, and feature selection. Ours operates on a single preprocessed post-contrast T1-weighted volume and requires no lesion delineation. This removes the expert-annotation burden and the associated inter-observer variability, and it avoids dependence on lesion contours that were not consistently suited to isolating enhancing metastases in this dataset. Whether that trade-off is worthwhile cannot be settled by the present data. Table 4 places the relevant studies side by side. The comparison is contextual only: the studies differ in cohort composition, class balance, input sequences, segmentation requirements, preprocessing, and validation design, and the reported metrics are therefore not directly comparable.

## 5. Limitations and Future Directions

Our study has several limitations. First, the model relies exclusively on T1CE MRI sequences and therefore cannot leverage complementary biological information available in modalities such as FLAIR, T2-weighted imaging, or DWI. Second, the dataset was retrospectively collected from a single center and was heavily dominated by 1.5 T acquisitions (220 vs. 19 scans at 3 T, 2 NA), limiting the feasibility of meaningful scanner-specific harmonization analyses and potentially affecting generalizability across imaging protocols and institutions. Third, the relatively small held-out cohort and substantial class imbalance, particularly for HER2-negative cases, resulted in wide confidence intervals and limited statistical stability of several reported metrics. Additionally, the model-development pipeline incorporated multiple optimization and selection stages, including hyperparameter optimization, ensemble selection, calibration, threshold tuning, and post-evaluation refinement of the ensemble aggregation strategy, increasing the possibility of optimism bias despite evaluation on a patient-independent held-out test set. Internal cross-validation on the development cohort may have introduced additional optimism during model selection because fold construction prioritized workable multilabel class distributions rather than patient-level separation, although the final held-out evaluation remained patient independent. We also did not benchmark the proposed encoder against alternative deep-learning architectures such as DenseNet, EfficientNet, or Vision Transformer-based models, nor did we conduct component-wise ablation analyses for data augmentation, Bayesian hyperparameter optimization, probability calibration, or the loss function, as these were incorporated in the pipeline based on their intended roles in small, imbalanced medical-imaging datasets. Fourth, another limitation is the absence of paired primary-tumor receptor status in our patient cohort. Related to this, receptor labels in the source dataset are recorded at the patient level, and the underlying histologic assessment was obtained variably from lymph node biopsy, the primary breast tumor, or a metastatic site that was not necessarily intracranial. The reference standard is therefore heterogeneous in origin and may not reflect the receptor status of the imaged brain metastasis. Lesion-specific annotations are also unavailable, so all scans from a given patient inherited a single ER, PR, and HER2 label. The model therefore estimates patient-level receptor status, and we could not assess whether receptor expression varied between individual metastases within a patient. Therefore, although receptor discordance is an important clinical motivation for this study, we could not evaluate whether the model performs differently in receptor-discordant cases. Future paired primary-metastatic datasets are needed to determine whether MRI-based receptor prediction can reliably identify clinically actionable discordance. Additionally, future models should incorporate FLAIR, T2-weighted imaging, and DWI/ADC to capture edema-related, peritumoral, and microstructural features that may be particularly important for hormone-receptor prediction. The modest cohort size limits generalization and stability assessment, and consequently, the reported findings should be interpreted as preliminary proof-of-concept results that require validation in larger multicenter cohorts.

## 6. Strengths of Our Study

Despite these limitations, this study also has notable strengths. We developed a mask-free DL model that uses a single MRI modality to estimate breast cancer receptor status, reducing dependence on manual segmentation and multimodal acquisition. This design also avoids dependence on lesion annotations that were not consistently suited to isolating enhancing metastases. Furthermore, we incorporated IG maps to improve methodological interpretability, providing voxel-level visualizations of the image regions contributing to model outputs, and enabling qualitative assessment of attribution plausibility.

## 7. Conclusions

In summary, this proof-of-concept study shows that a segmentation-free 3D CNN can be trained on preprocessed post-contrast T1-weighted MRI to recover some receptor-associated signal in BCBM. Discrimination was highest for PR, modest for HER2, and close to chance for ER, and every estimate was imprecise in a held-out cohort of 24 patients. These results do not establish clinical utility and do not support use of the model for receptor assessment in patient care. Receptor status guides systemic therapy in metastatic breast cancer, and discordance between primary tumors and brain metastases is common, so a validated non-invasive method of assessing intracranial receptor status would be clinically valuable. Our work provides a mask-free, single-sequence framework with built-in voxel-level attribution that can be evaluated at larger scale. Multicenter studies with substantially larger, patient-independent cohorts and multiparametric input, followed by prospective evaluation, are required before any diagnostic value can be claimed.

## Figures and Tables

**Table 1:** Class distribution of receptor statuses (ER, PR, HER2) across all 241 usable scans in the analysis cohort, including unknown labels.

Receptor	Positive	Negative	Unknown	Pos/Neg Ratio
ER	122 (50.6%)	114 (47.3%)	5 (2.1%)	1.070175
PR	79 (32.8%)	153 (63.5%)	9 (3.7%)	0.516340
HER2	141 (58.5%)	88 (36.5%)	12 (5.0%)	1.602273

**Table 2:** Hyperparameter Table.

Hyperparameter	Value		
Learning rate	3.43 × 10^−5^		
Weight decay	4.52 × 10^−4^		
Encoder depth	5 blocks		
MLP dropout probability	0.065		
	**ER**	**PR**	**HER2**
ASL positive focusing *γ*^(+)^	0.366	0.295	0.241
ASL negative focusing *γ*^(−)^	0.192	3.390	1.679
ASL positive shift *m*^(+)^	0.017	0.004	0.013
ASL negative shift *m*^(−)^	0.034	0.014	0.032

**Table 3:** Classification and diagnostic performance metrics of the proposed ensemble model on the held-out test set for ER, PR, and HER2 prediction. Confidence intervals (95%) for accuracy, PPV, sensitivity, specificity, and NPV were computed using Wilson score intervals. Confidence intervals (95%) for F1-score and AUROC were estimated using percentile bootstrap resampling (10,000 iterations).

Metric	ER	PR	HER2
Accuracy	0.5417 [0.3507–0.7211]	0.7917 [0.5953–0.9076]	0.7917 [0.5953–0.9076]
PPV/Precision	0.4667 [0.2481–0.6988]	0.6667 [0.3000–0.9032]	0.8571 [0.6536–0.9502]
Sensitivity/Recall	0.7000 [0.3968–0.8922]	0.5714 [0.2505–0.8418]	0.9000 [0.6990–0.9721]
Specificity	0.4286 [0.2138–0.6741]	0.8824 [0.6566–0.9671]	0.2500 [0.0456–0.6994]
NPV	0.6667 [0.3542–0.8794]	0.8333 [0.6078–0.9416]	0.3333 [0.0615–0.7923]
F1-Score	0.5600 [0.2857–0.7692]	0.6154 [0.2500–0.8889]	0.8780 [0.7568–0.9756]
AUROC	0.5714 [0.3264–0.7963]	0.7899 [0.5370–0.9773]	0.6375 [0.3361–0.9254]
Support (+)	10 (41.7%)	7 (29.2%)	20 (83.3%)
Support (−)	14 (58.3%)	17 (70.8%)	4 (16.7%)
Total Samples	24	24	24

**Table 4:** **Contextual comparison of MRI-based receptor-status prediction studies in breast cancer brain metastases.**

Study	Dataset and Validation Strategy	Input MRI Sequences	Model Type	Segmentation and Preprocessing Requirements	Reported Performance	Interpretation and Trade-Off
**Luo et al.** [11]	BCBM cohort; radiomics model with train/test evaluation	T1CE, T2WI, FLAIR	Handcrafted radiomics + machine learning	Required lesion segmentation, radiomic feature extraction, feature selection, and conventional ML classification	Reported high receptor-prediction performance, with test accuracies around 78–83% for ER, PR, and HER2	Stronger performance likely benefited from multisequence MRI and lesion-level radiomic features, but the workflow depends on segmentation and radiomics preprocessing
**Heitkamp et al.** [17]	BCBM cohort; radiomics-based validation strategy	T1CE, non-enhanced T1, FLAIR	Handcrafted radiomics + machine learning	Required quantitative feature extraction from metastases and multisequence imaging	Reported AUROCs approximately 0.73–0.82 across receptor targets	Demonstrated that receptor-associated imaging phenotypes, especially ER-related patterns, may rely on FLAIR-derived information; however, the approach remains preprocessing- and feature-engineering-dependent
**Christodoulou et al. (this study)**	TCIA BCBM-RadioGenomics dataset; patient-independent held-out test set of 24 patients	Post-contrast T1-weighted MRI only	Mask-free 3D ResNet ensemble with Integrated Gradients explainability	No manual segmentation used as model input; end-to-end mask-free DL pipeline using cropped whole-brain T1CE volumes	Test AUROC: ER 0.57, PR 0.79, HER2 0.64	The model offers greater scalability, and reduced dependence on manual segmentation and multimodal imaging; however, lower discrimination, particularly for ER, likely reflects the stricter T1CE-only input and absence of FLAIR/ADC information.

Footnote: Reported performance values are provided for contextual comparison only and should not be interpreted as a direct head-to-head ranking. Studies differed in cohort composition, sample size, receptor class balance, MRI sequences, segmentation requirements, preprocessing pipelines, model type, and validation strategy. The purpose of this table is to highlight methodological trade-offs between multisequence radiomics approaches and the present T1CE-only, mask-free deep learning framework.

## Data Availability

Publicly available datasets were analyzed in this study. The imaging and clinical data are available from The Cancer Imaging Archive Breast Cancer Brain Metastases Radiogenomics dataset: https://doi.org/10.7937/RRSE-W278. The code supporting the findings of this study is openly available at: https://github.com/Const1357/BCBM-RadioGenomics-Classifier.git (accessed on 25 June 2026).

## Abbreviations

The following abbreviations are used in this manuscript:

ADC	Apparent Diffusion Coefficient
AI	Artificial Intelligence
ASL	Asymmetric Focal Loss
AUC	Area Under the Curve
AUROC	Area Under the Receiver Operating Characteristic Curve
BCBM	Breast Cancer Brain Metastasis
BCE	Binary Cross-Entropy
CNN	Convolutional Neural Network
CNS	Central Nervous System
DL	Deep Learning
DWI	Diffusion-Weighted Imaging
ER	Estrogen Receptor
FLAIR	Fluid-Attenuated Inversion Recovery
GAP	Global Average Pooling
HER2	Human Epidermal Growth Factor Receptor 2
IG	Integrated Gradients
L-BFGS	Limited-memory Broyden–Fletcher–Goldfarb–Shanno
ML	Machine Learning
MLP	Multilayer Perceptron
MRI	Magnetic Resonance Imaging
NPV	Negative Predictive Value
OOF	Out-of-Fold
PPV	Positive Predictive Value
PR	Progesterone Receptor
ResNet	Residual Neural Network
ROC	Receiver Operating Characteristic
SRS	Stereotactic Radiosurgery
TCIA	The Cancer Imaging Archive
T1CE	Contrast-Enhanced T1-Weighted Imaging

## References

[1] Raghavendra A.S., Ibrahim N.K. (2024). Breast Cancer Brain Metastasis: A Comprehensive Review. JCO Oncol. Pract..

[2] Witzel I., Oliveira-Ferrer L., Pantel K., Müller V., Wikman H. (2016). Breast cancer brain metastases: Biology and new clinical perspectives. Breast Cancer Res..

[3] Burstein H.J., Somerfield M.R., Barton D.L., Dorris A., Fallowfield L.J., Jain D., Johnston S.R.D., Korde L.A., Litton J.K., Macrae E.R. (2021). Endocrine Treatment and Targeted Therapy for Hormone Receptor–Positive, Human Epidermal Growth Factor Receptor 2–Negative Metastatic Breast Cancer: ASCO Guideline Update. J. Clin. Oncol..

[4] Kuksis M., Gao Y., Tran W., Hoey C., Kiss A., Komorowski A.S., Dhaliwal A.J., Sahgal A., Das S., Chan K.K. (2021). The incidence of brain metastases among patients with metastatic breast cancer: A systematic review and meta-analysis. Neuro-Oncology.

[5] Shen Q., Sahin A.A., Hess K.R., Suki D., Aldape K.D., Sawaya R., Ibrahim N.K. (2015). Breast Cancer with Brain Metastases: Clinicopathologic Features, Survival, and Paired Biomarker Analysis. Oncologist.

[6] Niwińska A., Murawska M., Pogoda K. (2010). Breast cancer brain metastases: Differences in survival depending on biological subtype, RPA RTOG prognostic class and systemic treatment after whole-brain radiotherapy (WBRT). Ann. Oncol..

[7] Mintz A., Perry J., Spithoff K., Chambers A., Laperriere N., on behalf of the Neuro-oncology Disease Site Group of Cancer Care Ontario’s Program in Evidence-Based Care (2007). Management of Single Brain Metastasis: A Practice Guideline. Curr. Oncol..

[8] Ogiwara T., Nitta J., Fujii Y., Watanabe G., Kuwabara H., Agata M., Kobayashi H., Miyaoka Y., Kitamura S., Hanaoka Y. (2022). A preliminary study of the diagnostic efficacy and safety of the novel boring biopsy for brain lesions. Sci. Rep..

[9] Lüönd F., Tiede S., Christofori G. (2021). Breast cancer as an example of tumour heterogeneity and tumour cell plasticity during malignant progression. Br. J. Cancer.

[10] Kotecha R., Tonse R., Rubens M., McDermott M.W., Odia Y., Appel H., Mehta M.P. (2021). Systematic review and meta-analysis of breast cancer brain metastasis and primary tumor receptor expression discordance. Neuro-Oncol. Adv..

[11] Luo X., Xie H., Yang Y., Zhang C., Zhang Y., Li Y., Yang Q., Wang D., Luo Y., Mai Z. (2022). Radiomic Signatures for Predicting Receptor Status in Breast Cancer Brain Metastases. Front. Oncol..

[12] Cho S., Joo B., Park M., Ahn S.J., Suh S.H., Park Y.W., Ahn S.S., Lee S.-K. (2023). A Radiomics-Based Model for Potentially More Accurate Identification of Subtypes of Breast Cancer Brain Metastases. Yonsei Med. J..

[13] Gillies R.J., Kinahan P.E., Hricak H. (2016). Radiomics: Images Are More than Pictures, They Are Data. Radiology.

[14] Christodoulou R.C., Pitsillos R., Papageorgiou P.S., Petrou V., Vamvouras G., Rivera L., Papageorgiou S.G., Solomou E.E., Georgiou M.F. (2025). Artificial Intelligence in Glioma Diagnosis: A Narrative Review of Radiomics and Deep Learning for Tumor Classification and Molecular Profiling Across Positron Emission Tomography and Magnetic Resonance Imaging. Eng.

[15] Choi S., Lee Y., Lee M., Byon J.H., Choi E.J. (2025). Deep Learning-Based Recurrence Prediction in HER2-Low Breast Cancer: Comparison of MRI-Alone, Clinicopathologic-Alone, and Combined Models. Diagnostics.

[16] Taha B., Wu D., Sabal L., Kollitz M., Venteicher A., Watanabe Y. (2025). MRI Dataset of Metastatic Breast Cancer to the Brain with Expert-Reviewed Segmentations and Tumor-derived Radiomic Features (BCBM-RadioGenomics) (Version 1) [Dataset]. The Cancer Imaging Archive. https://www.cancerimagingarchive.net/collection/BCBM-RadioGenomics/.

[17] Heitkamp A., Madesta F., Amberg S., Wahaj S., Schröder T., Bechstein M., Meyer L., Broocks G., Hanning U., Gauer T. (2023). Discordant and Converting Receptor Expressions in Brain Metastases from Breast Cancer: MRI-Based Non-Invasive Receptor Status Tracking. Cancers.

[18] Paszke A., Gross S., Massa F., Lerer A., Bradbury J., Chanan G., Killeen T., Lin Z., Gimelshein N., Antiga L. (2019). PyTorch: An Imperative Style, High-Performance Deep Learning Library. arXiv.

[19] Simard P.Y., Steinkraus D., Platt J.C. (2003). Best practices for convolutional neural networks applied to visual document analysis. Seventh International Conference on Document Analysis and Recognition, 2003. Proceedings. Edinburgh, UK.

[20] He K., Zhang X., Ren S., Sun J. (2016). Deep Residual Learning for Image Recognition. 2016 IEEE Conference on Computer Vision and Pattern Recognition (CVPR), Las Vegas, NV, USA.

[21] Lin M., Chen Q., Yan S. (2014). Network In Network. arXiv.

[22] Ridnik T., Ben-Baruch E., Zamir N., Noy A., Friedman I., Protter M., Zelnik-Manor L. (2021). Asymmetric Loss For Multi-Label Classification. 2021 IEEE/CVF International Conference on Computer Vision (ICCV), Montreal, QC, Canada.

[23] Loshchilov I., Hutter F. (2019). Decoupled Weight Decay Regularization. arXiv.

[24] Akiba T., Sano S., Yanase T., Ohta T., Koyama M. (2019). Optuna: A Next-generation Hyperparameter Optimization Framework. arXiv.

[25] Ruopp M.D., Perkins N.J., Whitcomb B.W., Schisterman E.F. (2008). Youden Index and Optimal Cut--Point Estimated from Observations Affected by a Lower Limit of Detection. Biom. J..

[26] Simonyan K., Vedaldi A., Zisserman A. (2014). Deep Inside Convolutional Networks: Visualising Image Classification Models and Saliency Maps. arXiv.

[27] Niculescu-Mizil A., Caruana R. (2005). Predicting good probabilities with supervised learning. Proceedings of the 22nd International Conference on Machine Learning—ICML ’05, Bonn, Germany.

[28] Hou Y., Nitta H., Li Z. (2023). HER2 Intratumoral Heterogeneity in Breast Cancer, an Evolving Concept. Cancers.

[29] Guliyev M., Güren A.K., Özge E., Çolak R., Majidova N., Alkan Şen G., Safarov S., Günaltılı M., Fidan M.C., Gültürk İ. (2025). The Impact of Progesterone Receptor Status on Survival Outcomes in Metastatic Breast Cancer Patients Treated with First-Line CDK4/6 Inhibitors. Cancers.

[30] Ahn S.J., Park M., Bang S., Cho E., Ahn S.G., Suh S.H., Lee J.M. (2018). Apparent diffusion coefficient histogram in breast cancer brain metastases may predict their biological subtype and progression. Sci. Rep..

